# How common are foot problems among individuals with diabetes? Diabetic foot ulcers in the Dutch population

**DOI:** 10.1007/s00125-017-4274-7

**Published:** 2017-04-13

**Authors:** Robert M. Stoekenbroek, Joost L.C. Lokin, Mark M. Nielen, Erik S.G. Stroes, Mark J.W. Koelemay

**Affiliations:** 1 Department of Vascular Surgery, Academic Medical Center, University of Amsterdam, P.O. Box 22660, 1100 DD Amsterdam, the Netherlands; 2 Department of Vascular Medicine, Academic Medical Center, University of Amsterdam, Amsterdam, the Netherlands; 3 NIVEL (Netherlands institute for health services research), Utrecht, the Netherlands

**Keywords:** Diabetic foot, Screening, Ulcers

## Abstract

**Aims/hypothesis:**

Contemporary data on diabetic foot ulcer prevalence are scarce. Most studies were conducted in the 1990s, reporting incidence rates of 1.9–2.6%. Since then the prevalence of diabetes has doubled and the organisation of diabetes care has undergone major changes. Up-to-date data that quantify the occurrence of diabetic foot ulcers are required and could serve as baseline measures for future studies.

**Methods:**

Individuals with diabetes (*n* = 81,793) were identified from the NIVEL (Netherlands institute for health services research) Primary Care Database, which contains data for standardised routine care and is representative of the Dutch population. The annual incidence rates of ulcers and other foot abnormalities were calculated using data collected between 2010 and 2013. To account for inaccuracies, incidence rates were calculated using: (1) only individuals with a documented foot examination; (2) all individuals; and (3) individuals with explicit documentation of present/absent foot ulceration.

**Results:**

There were 412 individuals with documented ulceration during the registration period (0.50%). The annual incidence rate of foot ulcers was 0.34% (range 0.22–1.08%). Of those individuals with a documented foot examination, 14.6% had absent pedal pulsations, 17.3% had neuropathy and 10.1% had callus/pressure marks.

**Conclusions/interpretation:**

The annual incidence rate of foot ulcers in the current study was lower than previously reported. This observation could reflect the efficacy of screening practices and an increased awareness among professionals and patients. Nevertheless, approximately one in every five diabetic individuals had at least one identifiable risk factor on foot examination. This signifies the importance of preventive screening.

## Introduction

Diabetes is associated with an increased risk of peripheral sensory neuropathy, peripheral vascular disease and deformities of the feet. This combination may lead to the formation of foot ulcers, which require complex multimodal treatment including extensive local wound care, off-loading, revascularisation and optimisation of glycaemic control. Despite intensive therapy, only 60% of all diabetic foot ulcers are healed within 1 year of onset, and >10% of individuals with diabetic ulcers will eventually require lower extremity amputation [1, 2].

Contemporary data on the proportion of individuals with diabetes who develop foot ulcers are scarce. Some large population-based studies reported annual incidence rates of 1.9–2.6%. These studies, however, were primarily conducted in the 1990s [3–6]. Several developments may have substantially influenced the incidence of foot ulcers since those studies were performed. First, the prevalence of diabetes has doubled and the organisation of diabetes care has undergone major changes. Second, the severity of the problem is increasingly recognised, as evidenced by the publication of several position statements and treatment guidelines in recent years [7–10]. Third, individuals with diabetes, including those with diabetes-related complications, are increasingly treated in the primary care setting [11]. Up-to-date data could shine a light on the effects of these developments on ulcer incidence, and could assist in showing international differences and possible causes thereof. Furthermore, baseline measures could aid in estimating the effects of future developments and are important in determining optimal allocation of healthcare resources.

Therefore, the primary objective of this study was to obtain recent incidence and prevalence rates of diabetic foot ulcers in the Dutch population. Additionally, we studied the prevalence of risk factors for developing foot ulcers.

## Design and methods

### Data source

The Dutch NIVEL (Netherlands Institute for Health Services Research) Primary Care Database was used to obtain information on all individuals with diabetes registered at participating general practices [12]. This database holds longitudinal routine care data derived from electronic health records on, among others, morbidity, consultations, and physical and laboratory examination results for >1.5 million individuals in ~500 general care practices throughout the Netherlands. The database is representative of the Dutch general population. The electronic health records that serve as the source for the database use a standardised format. For example, morbidities and medication use are coded according to the International Classification of Primary Care (ICPC) and the Anatomical Therapeutic Chemical (ATC) classification system, respectively.

### Population

All individuals with type 1 or type 2 diabetes mellitus in the NIVEL Primary Care Database were included. The analysis was performed on data collected between 2010 and 2013.

As recommended by the International Working Group on the Diabetic Foot (IWGDF) and endorsed by the Dutch College of General Practitioners, GPs periodically perform a complete foot examination [7, 8]. A complete foot examination includes inspection for the presence of ulcers, pressure marks or callus, or previous amputation, palpation of the posterior tibial and dorsal pedal arteries, and sensory examination using a monofilament. The results of the examination are documented according to a standardised format in the electronic health records and could therefore be used in our analysis.

Dutch law allows the use of electronic health records for research purposes under certain conditions. According to this legislation, it is not obligatory to obtain informed consent from patients or approval by a medical ethics committee for observational studies that do not contain directly identifiable data (Dutch Civil Law, Article 7:458).

### Statistical analysis

The primary outcome measure was defined as the incidence rate of foot ulcers. Incidence rates were calculated by dividing the number of individuals with a new foot ulcer by the number of person-years at risk during the registration period. Two strategies were implemented to improve the accuracy of our data. First, to avoid overestimating the incidence rate due to the inclusion of individuals with prevalent ulcers, the first 3 months of the registration period were considered the ‘lead-in phase’ and individuals with an ulcer during this period were excluded from the analysis.

Second, we provide a range of incidence rates under different assumptions on the accuracy of the registration: (1) including only individuals for whom at least one component of the foot examination had been documented at any point during the observation period as proof that the foot has actually been examined; (2) including all individuals in the population in the denominator; and (3) including only those individuals for whom the absence or presence of ulcers was explicitly documented at any point during the observation period in the denominator. In addition, we performed a sensitivity analysis, which comprised only individuals from practices at which ≥70% of diabetic individuals underwent at least one complete foot exam during the study period.

Additionally, we calculated the prevalence of foot ulcers during the 4 years of observation (i.e. the period prevalence) by dividing the number of individuals with a recorded foot ulcer by the number of individuals for whom the results of at least one component of the foot examination had been documented during the entire registration period.

The prevalence of risk factors for developing foot ulceration was calculated by dividing the number of individuals with absent pedal artery pulsations, pressure marks/callus or abnormal findings on the monofilament examination by the number of individuals for whom the results of at least one component of the foot exam was documented.

Continuous values are expressed as mean and SD, or median and interquartile range (IQR) where appropriate. Dichotomous values are presented in absolute numbers and percentages. Statistical analyses were performed using SPSS software version 22.0 (Chicago, IL, USA).

## Results

### Study population

Data were available from 355 general care practices, comprising 81,793 individuals with diabetes. The total number of person-years of follow-up was 195,806 years. The characteristics of the study population are listed in Table 1. A complete foot examination during the observation period was documented in 46,087 (56.3%) individuals, whereas at least one component of the foot examination was documented in 52,524 (64.2%) individuals. The number of practices at which ≥70% of individuals with diabetes had at least one documented foot examination was 132 (37.2%).

**Table 1:** Population characteristics (*N* = 81,793)

Characteristic	
Male sex	41,689 (51.0)
Age (years)	65.8 ± 14.4
BMI (kg/m^2^)	29.9 ± 5.5
Duration of diabetes (years)	4.3 (1.1–8.5)
Smoking	
Never	28,381 (34.7)
Current	11,491 (14.0)
Former	20,859 (25.5)
Not documented	21,052 (25.7)
Peripheral arterial disease	6501 (7.9)
Previous amputation	826 (1.0)
Previous stroke	5470 (6.7)
HbA_1c_ (%)	6.6% (6.2–7.4)
HbA_1c_ (mmol/mol)	49 (44–57)
Insulin usage	19,590 (24.0)
Lipid-lowering drugs	56,652 (69.3)
Antihypertensive drugs	59,442 (72.7)
GFR (<60 ml min^−1^ [1.73 m]^−2^)	6168 (7.5)

Data are presented as *n* (%), mean ± SD or median (IQR)

Active ulceration during the study period was recorded in 412 individuals (0.5%). In total, 29 individuals had a documented ulcer during the first 3 months of registration and were excluded from the incidence analysis. The total number of person-years at risk was 174,921, and the total number of new cases of foot ulcers was 383. The annual incidence rate among individuals with at least one documented component of the foot examination was 0.34% (Table 2). Under the assumption that all ulcers were documented, the annual incidence rate was 0.22%; whereas the incidence rate would be 1.08% if only the 14,421 individuals with explicit documentation of the absence or presence of foot ulceration were included (Table 2).

**Table 2:** Incidence and prevalence rates

	At least one component documented^a,b^	All individuals^c^	Ulcer status explicitly documented^d^
Person-years at risk	112,942	174,921	35,302
Annual incidence rate (%)	0.34	0.22	1.08
Four year prevalence (%)	0.78	0.50	2.85

^a^At least one component of the standard foot examination was documented

^b^ *n* = 52,495 for incidence and 52,524 for prevalence

^c^ *n* = 81,764 for incidence and 81,793 for prevalence

^d^ *n* = 14,421 for incidence and 14,450 for prevalence

In the sensitivity analysis, which included only the 132 practices at which ≥70% of individuals had undergone at least one complete foot exam, the annual incidence rate was 0.27%.

Given the 412 individuals with active ulceration during the registration period, the 4 year prevalence among all individuals with at least one documented component of the foot examination was 0.78% (Table 2). Under the assumption that all ulcers were recorded, the corresponding prevalence rate would be 0.50%; whereas the 4 year prevalence rate would be 2.85% if only individuals with explicit documentation of the absence or presence of foot ulceration were included.

Approximately 20% of all individuals had at least one abnormality on foot examination (Table 3). Sensory neuropathy was the most commonly encountered risk factor.

**Table 3:** Abnormalities on foot examination

Abnormality	At least one component documented^a^ (*n* = 52,524)	All individuals (*n* = 81,793)
Absent pedal pulsations	7652 (14.6)	7652 (9.4)
Sensory neuropathy	9075 (17.3)	9075 (11.1)
Callus/pressure marks	5295 (10.1)	5295 (6.5)
Any abnormalities	16,573 (31.6)	16,573 (20.3)

Data presented are *n* (%)

^a^At least one component of the standard foot examination was documented

## Discussion

In this study of 81,793 individuals with diabetes in the Dutch primary care setting, the annual incidence of foot ulceration during the 4 years of observation was estimated at 0.34% (0.22–1.08% under different assumptions of the accuracy of registration). Despite the relatively low incidence and prevalence rates, >20% of individuals with diabetes had at least one identifiable risk factor for ulceration on foot examination. This finding signifies the importance of screening, as these individuals have a markedly increased risk of developing foot ulcers.

### Time trends

Two previous Dutch population studies have reported foot ulcer rates among individuals with diabetes [6, 13]. In a prospective study in the primary care setting between 1993 and 1998, Muller et al reported a mean annual ulcer incidence of 2.1% [6]. Similarly, de Sonnaville et al performed annual foot examinations of 609 individuals with diabetes from 22 general practices between 1992 and 1995 and found a point prevalence for foot ulceration of 1.8% [13]. Although the limited number of studies precludes definitive conclusions on time trends, our results suggest that it is unlikely that the large increase in the prevalence of diabetes and the changes in diabetes care have been accompanied by an increased occurrence of foot ulcers among the diabetes population in the Netherlands.

### International comparisons

International population-based studies have reported higher foot ulceration rates than those found in our population. These studies were primarily conducted in the 1990s. The UK-based North-West Diabetes Foot Care Study reported an annual incidence of diabetic foot ulceration of 2.2% during 2 years of follow-up between 1994 and 1996 [4]. Compared with our study, individuals had a longer duration of diabetes (8.9 vs 4.3 years) and more frequent absent pedal artery pulsations (21.1% vs 12.1–19.3%). More recently, in 2008–2009, Hurley et al performed foot screenings of 563 individuals with diabetes from general practices in the West of Ireland Diabetes Foot Study [5]. They reported an annual incidence rate of 2.6%. Again, population characteristics could have contributed to the differences between findings. For example, in our study there were fewer men (51% vs 60%), mean HbA_1c_ values were lower (6.6% vs 7.3% [49 vs 56 mmol/mol]) and individuals had a shorter duration of diabetes (4.3 vs 7.7 years). In the early 1990s, Ramsey et al determined the incidence of foot ulcers in the USA using a retrospective database study of 8905 individuals with diabetes [3]. The cumulative 3 year incidence in this study was 5.8%, for an annual incidence of 2.0%. However, it is not possible to speculate on the possible factors contributing to the differences in incidence rates since few details are given on population characteristics for this study.

Similarly, studies that only report prevalence rates have provided estimates that are relatively high compared with the present study. For example, Manes et al reported a point prevalence for foot ulceration of 4.8% in 821 diabetic individuals from Greece, and a population-based database study from Spain comprising 149,015 individuals found a 1 year prevalence rate of 1.9% [14, 15]. Again, possible causative factors for the differences in reported rates cannot be studied since few details are given on the population characteristics in either study.

Of note, the UK National Diabetes Audit, which reports on the proportion of individuals with diabetes receiving care according to national recommendations, reported that 87% of patients received a foot examination [16]. This figure compares favourably with the 56% found in our study. One possible explanation for the difference might be a more complete registration in the UK. However, it is also possible that higher screening rates are a result of a national initiative in the UK to improve diabetes care, i.e. the National Service Framework for Diabetes [17].

An important observation in the present study is that despite the relatively favourable estimates of ulcer incidence, identifiable risk factors were quite prevalent. In line with the overall lower rates of actual ulceration, these numbers compare relatively favourably with previous studies. It could be speculated that the introduction of integrated care in general practices has resulted in earlier identification of individuals who are at risk of developing diabetic ulcers and more effective prevention. In line with this hypothesis, it appears that our population was healthier compared with previous studies (e.g. lower incidences of peripheral arterial disease and stroke), although differences in definitions and registration methods makes actual comparison difficult. One could expect higher reported prevalence rates for risk factors with improved screening and awareness of diabetic foot problems. However, screening would be expected to reduce ulcer incidence by enabling earlier preventive intervention among individuals with an increased ulcer risk. Interventions include shoe modifications, targeted education on foot care and pedicures.

To the best of our knowledge, this is the first study to report the prevalence of foot ulcers in individuals with diabetes in the Netherlands since the 1990s. A major strength of this study is the fact that we were able to use a nationwide sample of 81,793 individuals representative of the Dutch diabetes population. Nevertheless, a major disadvantage is the lack of completeness of registration, which is inherent to using large routine care databases. Approximately 36% of the diabetic individuals in the database did not have documented results of a foot examination. It is likely that the majority of these individuals for whom no examination was performed represented a low risk of ulceration. However, it is also possible that the registration itself is incomplete. We addressed this issue by providing a range of incidence estimates. Individuals for whom at least one component of the foot exam was documented were used to provide the best estimate, because the presence of these data suggests that any ulcer would have been documented as well. It is theoretically possible that some individuals may not have been registered because they entered secondary care without consulting a GP. However, because a GP is generally required in the Netherlands, we believe that the number of individuals missed in the registration due to self-referral and the consequent impact on our results and conclusions is limited. To provide an accurate estimate on the incidence of ulcers, we excluded individuals who had an existing ulcer during the first 3 months of the registration. However, we cannot exclude the possibility that some individuals were inappropriately documented as having an incident ulcer. However, the low number of individuals with ulcers during the first 3 months of the registration suggests that any misclassification will probably not have materially affected the estimates. Furthermore, we plotted the proportion of individuals free of ulcers against time from the start of the registration. Identification of pre-existing ulcers would be expected early in the registration period; however, ulcer occurrence was roughly evenly distributed across the course of the registration. Finally, individuals who do not seek the care of a GP have not been included in this analysis, which may have resulted in an underestimation of the number of ulcers.

In conclusion, the annual incidence rate of foot ulcers in the current study was lower than previously reported. This observation could reflect the efficacy of screening practices and an increased awareness of foot problems in individuals with diabetes. Nevertheless, approximately 20% of diabetic individuals had at least one identifiable risk factor on foot examination signifying the importance of preventive screening.

## Abbreviation

NIVEL: Netherlands Institute for Health Services Research
